# CEO financial background, managerial ownership, and corporate innovation: Insights from imprinting theory

**DOI:** 10.3389/fpsyg.2023.1126853

**Published:** 2023-02-14

**Authors:** Yu Gao, Yahao Tang, Jiruo Zhang

**Affiliations:** Department of Accounting, Qingdao University, Qingdao, China

**Keywords:** CEO financial background, corporate innovation, managerial ownership, imprinting theory, corporate behavior

## Abstract

The global ecological environment is facing increasingly severe challenges; therefore, it is crucial to implement sustainable development policies and promote corporate innovation. Based on imprinting theory, we examine the relationship between CEO financial background and corporate innovation within the Chinese context. The results confirm that CEOs with a financial background negatively impact corporate innovation, while managerial ownership mitigates this effect. Existing literature has considered the impact of CEO background on corporate innovation; however, it mainly takes up corporate innovation from the perspective of upper-echelon theory. In addition, the mechanism of CEO financial background on corporate innovation is ambiguous in the Chinese cultural context. This study enriches the literature on the relationship between the characteristics of CEO background and corporate behavior, thereby offering guidance for corporate innovation practices.

## 1. Introduction

In February 2021, the United Nations Environment Programme issued the report *Making Peace with Nature*. In the report, climate change, natural degradation and biodiversity loss, and pollution and waste crisis are identified as the three major environmental crises that the world is currently facing. These crises pose a serious threat to human survival. For instance, air pollution causes 300,000 to 700,000 premature deaths each year, while 25 million cases of chronic laryngitis among children are reported yearly. Further, in 2022, major ecological events such as extremely high temperatures in Europe and volcanic eruptions in Japan occurred. As these ecological and environmental problems aggravate, humanity’s future is at risk; consequently, the idea of sustainable development is progressively becoming a worldwide objective. As the world’s largest carbon emitter and energy consumer (Zhang and Chen, 2021), China announced during the 2014 *U.S.-China Joint Announcement on Climate Change* that its carbon dioxide emissions would peak by 2030 and that it would strive to achieve carbon neutrality by 2060. Simultaneously as the world’s second largest economy and the largest contributor to world economic growth, China’s realization of sustainable development would have a global impact.

In order to achieve the “double carbon” target and promote sustainable development strategies, higher standards for China’s economic transformation, technical innovation, and capital investment have been put forward (Wang et al., 2021). Sustainable development is closely tied to economic, ecological, and social development, which are all reliant on the accessibility of technologies, innovative tactics, and favorable governmental policies (Vollenbroek, 2002). Therefore, technological innovation plays a key role in sustainable economic development. Schumpeter and Backhaus (2003) noted that enterprises play a vital role in technological innovation for economic development. Enterprises are the backbone of the national economy and a crucial driving force of technological innovation; improving their independent innovation capability enables breakthroughs in core technological problems, thereby promoting the transformation and upgrading of the industrial structure and sustainable development. Therefore, promoting technological innovation in Chinese enterprises has become an issue of great practical significance.

Scholars have studied the factors that influence firms’ technological innovation at the macro, middle, and micro levels. At the macro level, scholars have studied the macroeconomic environment (Lou et al., 2022) and intellectual property rights protection (Fang et al., 2017). At the middle level, previous studies have analyzed factors such as financial analyst coverage (Guo et al., 2019) and market competition (Yang M. J. et al., 2021). At the micro level, scholars have studied CEO traits’ impact on corporate innovation, primarily from the perspective of their foreign (Yuan and Wen, 2018), military (Benmelech and Frydman, 2015), and marketization experience (Hou et al., 2021). While existing studies have considered the impact of CEO characteristics on corporate innovation, they have some limitations. First, existing literature mainly takes up this relationship using upper-echelon theory and does not fully explain the internal mechanism of the impact of CEO characteristics on corporate innovation. Second, few studies have considered the impact of CEO financial background (FB) on corporate innovation, and the conclusions of these studies are controversial. Third, existing literature is mostly rooted in the western context, and little work has been done in the Chinese cultural context.

A distinctive phenomenon has evolved as a result of the growth of China’s capital market: many people with a background in finance have entered non-financial industries, later becoming executives. Therefore, it is becoming significantly more important to explore the relationship between CEO FB and corporate innovation. Two key questions to address are “Can CEO FB affect corporate innovation?” and “What are the influencing factors of this relationship?”

Based on imprinting theory, this study used data from Chinese A-share-listed enterprises for the period 2017–2021 as the research sample to analyze the impact of CEO FB on corporate innovation in the Chinese context. The data on individual CEOs and firms were gathered from the China Stock Market and Accounting Research Database (CSMAR), whereas data on corporate innovation were collected from the Chinese Research Data Service (CNRDS). We found that CEOs with FB had a negative impact on corporate innovation through cognitive imprinting and capability imprinting. Additionally, when CEOs have higher managerial ownership, the negative effect of their FB on corporate innovation will be mitigated. This study’s findings were found to be robust.

The present study made the following contributions: First, the existing literature is extensive, remaining firmly rooted on upper-echelon theory and mainly paying attention to the statistical relationship between CEO experience or background and corporate innovation. However, prior studies have overlooked this relationship’s intrinsic mechanisms. Based on imprinting theory, we provide an in-depth interpretation of the mechanisms by which CEO characteristics influence corporate innovation. Second, prior research on CEO background has mainly considered their foreign (Yuan and Wen, 2018), military (Benmelech and Frydman, 2015), and academic experience (Shen et al., 2020), and little attention has been paid to their financial experience. This study incorporates CEO FB into the analytical framework of corporate innovation and adds managerial ownership as a moderating variable, thus providing new empirical evidence for the study of factors that influence corporate innovation. Third, several studies (Hirshleifer et al., 2012; Bromiley and Rau, 2016; Kashmiri et al., 2017) have examined the Western context, but the cognition and behavior of CEOs are deeply influenced by culture. Therefore, we explored the mechanisms from the perspective of Chinese culture, thus expanding the applicability of the relevant literature.

The remainder of this paper is organized as follows. (Section “2. Literature review and hypothesis development”) summarizes the available research and presents the research hypothesis. (Section “3. Research design”) presents the research design, thereby introducing the data sources, sample selection, variable measurement, and establishment of the empirical model. The findings of the empirical analysis are presented in (Section “4. Results”), together with descriptive statistics, correlation analysis, main regression analysis, and the outcomes of the robustness test. The research findings and implications are presented in (Section “5. Conclusion and discussion”).

## 2. Literature review and hypothesis development

### 2.1. Literature review

Innovation is a prominent component affecting the economic development of the country and the firm (Fagerberg, 2004). Numerous studies have attempted to explore the factors that influence firm innovation at the macro, middle, and micro levels. At the macro level, several studies have highlighted factors associated with corporate innovation from the perspectives of the institutional environment, national policies, and the social legal system (Fang et al., 2017; Lou et al., 2022). Moreover, there has been extensive research on mid-level influencing factors, such as the impact of financial analyst coverage (Guo et al., 2019), market competition (Yang M. J. et al., 2021), and other factors on firm innovation. At the micro level, researchers attempted to evaluate the impact of ownership structure (Minetti et al., 2015), corporate policy (Krammer, 2022), and CEO political preference (Han, 2019) on corporate innovation.

R&D innovation is a complex and risky investment decision. Simultaneously, CEOs are the most critical decision-makers in firms; accordingly, CEO characteristics have a significant impact on firms’ innovation decisions. Prior studies classify CEO characteristics into personality and background traits. In terms of personality traits, using data from the U.S., Hirshleifer et al. (2012) discovered that overconfident CEOs increase companies’ revenue elasticity; thus, when they have sufficient cash, their willingness and drive to innovate increase. In a follow-up study, Abdel-Khalik (2014) found a significant positive relationship between CEO risk tolerance and firm innovation activities. Additionally, several studies on CEO characteristics have focused on narcissism (Kashmiri et al., 2017), proactiveness (Kiss et al., 2022), overconfidence (Pitenoei et al., 2019), hometown identity (Ren et al., 2021), sensation seeking (Sunder et al., 2017), among other characteristics. Regarding CEO background characteristics, several factors have been investigated, such as age (Chowdhury and Fink, 2017), gender (Strohmeyer et al., 2017), tenure (Cucculelli, 2018), compensation (Mazouz and Zhao, 2019), executive change (Biscotti et al., 2018), education level (Bromiley and Rau, 2016), expertise (Tabesh et al., 2019), and social background (Liu et al., 2018). Furthermore, previous studies have also explored CEOs’ foreign, military, academic, marketization-related, and diversified experience (Benmelech and Frydman, 2015; Chen et al., 2015; Yuan and Wen, 2018; Shen et al., 2020; Hou et al., 2021). In a study investigating CEO background characteristics, according to Gao and Hafsi (2015), executives with experience in manufacturing, research and development (R&D), and design functions are more knowledgeable about the entire R&D process, which increases the likelihood of R&D success. CEO individual characteristics impact corporate technological innovation, though the impact varies remarkably.

Prior findings on the relationship between CEO FB and corporate innovation have been inconsistent and contradictory. As previously described, there are three main perspectives on the relationship between CEO FB and corporate innovation: positive, negative, and unrelated. Firstly, some scholars argue that CEO FB is beneficial to corporate innovation. In the context of European countries, by creating new information exchange channels with financial institutions, Cosci et al. (2016) researched how CEOs with FB can reduce information asymmetry and enhance firms’ innovation performance. Furthermore, Li et al. (2021) reported that, within the context of U.S. enterprises, executives with FB are conducive to reducing corporate investment inefficiencies. Conversely, some scholars have reported no positive correlation between CEO FB and corporate innovation. According to data gathered from publicly listed Chinese companies, Liu et al. (2020) made the case that firms with more financial-background executives have higher investments in financial assets, smaller investments in fixed assets, lower R&D investments, and more severe financial constraints, all of which are detrimental to innovation through the crowding-out effect. According to Yang C. et al. (2021), research on Chinese listed companies discovered that corporate innovation is negatively impacted by the crowding-out effect of increased financial investment, which is generated by the consistent behavior of CEOs with financial expertise. Analyzing U.S. firms, Custódio and Metzger (2014) argued that financial-background CEOs have a considerable negative impact on firm innovation. In addition to the aforementioned literature, some scholars argue that there is no correlation between CEO FB and firm innovation. A significant analysis and discussion on the subject was presented by Barker and Mueller (2002), who obtained corporate data from *Business Week* and discovered that corporate R&D investment is the same regardless of whether the firm employs CEOs with FB, thus demonstrating that CEOs with FB are not correlated with corporate R&D investment.

Although the relationship between CEO FB and corporate innovation has been discussed in the literature, further research is necessary. First of all, a large and growing body of literature has investigated the impact of CEO FB based on upper-echelon theory, focusing more on the statistical link between CEO background and its economic consequences, without opening the “black box” and lacking in-depth analysis of the mechanism of this relationship. Moreover, while prior studies have mentioned the association between CEO FB and corporate innovation, there is no consensus; thus, further research is required to provide new evidence. Finally, most prior studies have examined the Western context; however, culture is a significant factor, so the same background may have diverse effects on people’s cognition and behavior in different cultural contexts. Hence, it is essential to analyze in the Chinese cultural context.

### 2.2. Hypothesis development

According to imprinting theory, during individuals’ formative professional years, work experience leaves an imprint on their psyche (especially on their cognition and capability), which will have a continuous impact on their career (Mathias et al., 2015). The financial industry is a high-risk sector (Calluzzo and Dong, 2015) with a distinct mode of operation and intensely stressful environment; thus, time spent in this sector is a critical period for CEOs. During this period, CEOs’ cognition and capability profiles are shaped to fit the financial work environment, which has a significant impact on the decisions they make later in their careers.

In terms of cognition, the CEO preference for short-term investments is determined by their financial experience. Due to its brief history, compared with Western economies, China’s capital market may exhibit speculative traits (Liu and Shrestha, 2008). Executives with FB are susceptible to the effects of the environment while working and studying the capital market. Consequently, their cognitive and behavioral patterns will be imprinted with speculation, which will shape their short-term speculative preference. Specifically, when making decisions, executives with a shorter time horizon are more inclined to focus on investment projects that can improve corporate performance quickly in the current environment (Stein, 1989; Laverty, 1996). Throughout their careers, these executives maintain the mindset and working practices they developed during this critical time (Dokko et al., 2009). Corporate innovation activities are risky, long-term (Zaheer et al., 1999), unclear about future growth (Pindyck, 1982), and capital-intensive. Once a company’s innovation activities fail, it becomes exposed to operational risks; therefore, CEOs with a short-term investment preference are more likely to pursue short-term/high-return projects when making investment decisions (Narayanan, 1985; Stein, 1988; Holmström, 1999), while abandoning long-term R&D projects with potentially higher yields.

In terms of capability, CEOs’ financial investment ability and social connections in finance lead to a “crowding-out effect” on corporate physical assets investment. CEOs with FB have a deeper and more specialized understanding of decision-making in the financial field (Graham et al., 2013), and their strengths lie in their mastery of financial instruments and knowledge of the financial industry. According to Waller et al. (1995), only corporate executives with professional knowledge and background can make decisions in a professional capacity. Executives with FB are capable of information processing, opportunity screening, and capital operation regarding finance; therefore, they are more inclined to make financial investments, while their companies become more financialized (Du et al., 2019). Additionally, CEOs with a background in finance can more quickly access investment opportunities by exploiting the social networks they are already a part of. Financial investments displace funds that would otherwise be available for R&D and other physical assets investments (Orhangazi, 2008), causing firms to use fewer resources for innovation as a result of financing restrictions, which will ultimately impede corporate innovation (Wang et al., 2017).

In summary, CEOs with an FB have a negative impact on corporate innovation in terms of both cognition and capabilities. Therefore, we propose hypothesis H1:

H1: CEOs’ FB has a negative impact on corporate innovation.

According to Palia and Lichtenberg (1999), the separation of ownership and control rights could lead to an interest conflict between shareholders and executives by giving self-interested executives an incentive to neglect their duties or use corporate resources to further their personal interests at shareholders’ expense (Jensen and Meckling, 1976). According to Baer (2012), innovation contributes to the growth of a firm’s competitive advantage and fosters corporate growth. Consequently, shareholders prefer firms that invest in R&D initiatives that will boost long-term profits. However, because investing in innovation is a high-risk, capital-intensive, and long-term corporate activity, executives may be tempted to reduce R&D investment to maximize their own profits. This has a detrimental effect on firms’ strategic planning and future growth (Cohen et al., 2013).

According to Jensen and Meckling (1976), there is an incentive compatibility effect as managerial ownership rises because it tends to bring management and shareholder interests closer together and lowers the principal-agent cost. Simultaneously, the corporate share structure changes, and the CEO’s share of corporate revenue shared in innovation activities increases. Therefore, executives are more likely to invest in projects that will maximize the value of the company when their interests are more strongly associated with those of the shareholders (Morck et al., 1988). Further, CEOs’ interests become increasingly aligned with those of the shareholders the more shares they own; consequently, they become more concerned about the long-term development of the firm and more likely to make innovative investments to enhance the enterprise value (Lin et al., 2011), thus mitigating the detrimental impact of their FB on corporate innovation. Therefore, the higher the managerial ownership, the more likely the negative impact of their FB on corporate innovation will be mitigated.

In sum, managerial ownership mitigates the negative impact of CEO FB on corporate innovation. Accordingly, we propose research hypothesis H2:

H2: Managerial ownership mitigates the negative impact of CEOs’ FB on corporate innovation.

## 3. Research design

### 3.1. Data and sample selection

This study examined data from Chinese A-share listed companies for the period between 2017 and 2021. CEO FB data were obtained from the CSMAR’s China Corporate Figure Characteristic Series. Missing data on CEO FB were supplemented by manually searching for the information, and we removed data that were still vacant. The data on corporate patents were collected from the CNRDS, which is widely used by Chinese scholars. The other data used in the model were all from the CSMAR.

The sample selection criteria were as follows: (i) excluding the financial industry samples; (ii) excluding the ST and *ST companies; (iii) excluding missing and abnormal samples of relevant data; (iv) excluding the current year listed company sample; (v) we applied 1 and 99% tailing to all continuous variables in the model to reduce the impact of an extreme value on the research results. Our final sample included unbalanced panel data of 12,248 firm-year observations.

### 3.2. Variable definitions

#### 3.2.1. Measurement of CEO FB

Referring to Yang C. et al. (2021) and Yang and Shen (2022), we defined CEO FB as CEOs with previous working experience in financial institutions, including commercial banks, investment banks, insurance companies, securities companies, securities registration and settlement companies, futures companies, trust companies, investment management companies, and exchanges. Accordingly, we constructed a dummy variable to capture whether the CEO has financial working experience. The value is 1 when the CEO has FB; otherwise, it is 0.

#### 3.2.2. Measurement of corporate innovation

Following previous studies (e.g., Liu et al., 2020; Pang and Wang, 2020; Lou et al., 2022), we measured corporate innovation by the number of firms’ patents, Chinese patents were divided into three types: invention patents, utility model patents, and design patents. We quantified innovation (PAT) by adding one to the total number of patent applications and used the natural logarithm of the result. Because earlier research has demonstrated that the application is more closely related to the actual period of innovation, we dated our patent data using the application year rather than the grant year (Griliches et al., 1986).

#### 3.2.3. Moderating variables

Referring to Li et al. (2007), managerial ownership (MO) is equal to the number of shares held by the CEO divided by the total number of shares.


M⁢O=S⁢h⁢a⁢r⁢e⁢s⁢h⁢e⁢l⁢d⁢b⁢y⁢t⁢h⁢e⁢C⁢E⁢O/t⁢o⁢t⁢a⁢l⁢s⁢h⁢a⁢r⁢e⁢s

#### 3.2.4. Control variables

Referring to Liu et al. (2020), Ren et al. (2021), and Yang C. et al. (2021), we selected the following control variables: leverage ratio (LEV), firm size (SIZE), firm age (FAGE), sales growth rate (SALEGRO), return on assets (ROA), Tobin’s Q, enterprises’ property right (SOE), the proportion of institutional investors holding (INSHOLD), the ratio of independent directors (IDRATIO) and board size (BSIZE), industry dummy (IND) and year dummy (YEAR).

Dividing total liabilities by total assets results in LEV. SIZE is the natural logarithm of total assets. We used the natural logarithm to calculate FAGE by adding one to the gap between the year the data were collected and the year it was founded. By dividing the growth in sales from the current year by that of the previous year, SALEGRO was calculated. The proportion of current net income to total assets was used to calculate ROA. The market value of assets divided by the book value of all assets results in Tobin’s Q. We determined SOE according to the type of actual controller; we assigned state-owned enterprises a value of 1; other types of enterprises were given a value of 0. Using the shares held by institutional investors relative to all shares, INSHOLD was calculated. The percentage of independent directors to all directors was used to calculate IDRATIO. The total number of directors on the board was BSIZE. In order to control the differences between industries and years, IND and YEAR were selected as the control variables. The industrial dummy variables were divided into industries according to the 2012 industry classification standard of China Securities Regulatory Commission. The manufacturing industry was categorized into the secondary industry classification, and the other industries were categorized into the primary industry classification. Table 1 presents the variable definitions.

**TABLE 1:** Variable definitions.

Abbreviation	Variables	Definition
FB	CEO financial background	If the CEO has a financial background, value is 1; otherwise, it is 0.
PAT	Corporate innovation	Ln (the total number of patents +1)
MO	Managerial ownership	Shares held by the CEO/total shares
LEV	Leverage ratio	Total liabilities/total assets
SIZE	Firm size	Ln (total assets)
FAGE	Firm age	Ln (the difference between the current year and the establishment year +1)
SALEGRO	Sales growth rate	Growth of current year’s sales revenue/total sales revenue of last year
ROA	Return on assets	Net income/total assets
Tobin’s Q	Tobin’s Q	The market value of the asset/the book value of the asset
SOE	Enterprises property right	Dummy variable that is 1 a firm has state-owned actual controllers and 0 otherwise.
INSHOLD	The proportion of institutional investors holding	Shares held by institutional investors/total shares
IDRATIO	The ratio of independent directors	Number of independent directors/total number of directors
BSIZE	Board size	Number of directors on the board
IND	Industry dummy variable	The industrial dummy variables were divided into industries according to the 2012 industry classification standard of the China Securities Regulatory Commission
YEAR	Year dummy variable	Value of 1 if it belongs to the year, otherwise 0

### 3.3. Empirical model

H1 predicted that CEO FB would have a negative impact on corporate innovation. The model was set using the ordinary least squares method to test H1. If the regression coefficient β_1_ of FB is negative and significant, H1 is supported.


$$P\,A\,T=\beta_{0}+\beta_{1}\,F\,B+\beta_{2}\,L\,E\,V+\beta_{3}\,S\,I\,Z\,E+\beta_{4}\,F\,A\,G\,E$$

$$\;\;+\beta_{5}\,S\,A\,L\,E\,G\,R\,O+\beta_{6}\,R\,O\,A+\beta_{7}\,T\,o\,b\,i\,n^{\prime }\,s\,q$$

$$\;\;+\beta_{8}\,S\,O\,E+\beta_{9}\,I\,N\,S\,H\,O\,L\,D+\beta_{10}\,I\,D\,R\,A\,T\,I\,O$$

$$\;\;+\beta_{11}\,B\,S\,I\,Z\,E+\Sigma \,I\,N\,D+\Sigma \,Y\,E\,A\,R$$

   +ε⁢(M⁢o⁢d⁢e⁢l⁢ 1)

H2 predicted that managerial ownership mitigates the negative effect of CEO FB on firm innovation. To test H2, we added the interaction term FB*MO based on model 1 to form model 2. If the coefficient β_2_ of FB*MO is positive and significant, H2 is supported.


$$P\,A\,T=\beta_{0}+\beta_{1}\,F\,B+\beta_{2}\,F\,B*M\,O+\beta_{3}\,M\,O+\beta_{4}\,L\,E\,V$$

$$\;\;+\beta_{5}\,S\,I\,Z\,E+\beta_{6}\,F\,A\,G\,E+\beta_{7}\,S\,A\,L\,E\,G\,R\,O+\beta_{8}\,R\,O\,A$$

$$\;\;+\beta_{9}\,T\,o\,b\,i\,n^{\prime }\,s\,q+\beta_{10}\,S\,O\,E+\beta_{11}\,I\,N\,S\,H\,O\,L\,D$$

$$\;\;+\beta_{12}\,I\,D\,R\,A\,T\,I\,O+\beta_{13}\,B\,S\,I\,Z\,E+\Sigma \,I\,N\,D+\Sigma \,Y\,E\,A\,R$$

   +ε⁢(M⁢o⁢d⁢e⁢l⁢ 2)

## 4. Results

### 4.1. Descriptive statistics

Table 2 shows the descriptive statistics of our sample. The average of FB is 0.06, indicating that 6% of CEOs have a background in finance. The average of PAT is 1.90, the minimum value is 0.00, and the maximum value is 9.35, with a standard deviation of 1.65, indicating a large variation in patent applications across firms. Regarding the control variables, the average ROA is 0.04, the minimum is −0.34, and the maximum is 0.21, indicating a large variation in profitability across firms. The average of LEV is 0.39, the minimum value is 0.06 and the maximum value is 0.86. The average SIZE is 22.19, and the standard deviation is 1.30. The average SOE is 0.26, indicating that 26% of the sample enterprises belong to state-owned enterprises. The average FAGE is 2.95. The average INSHOLD is 0.40. The average SALEGRO is 0.17, and the median is 0.12. The average of Tobin’s Q is 1.89. The average of IDRATIO is 0.38, indicating that 38% of the directors are independent and overall, the Chinese Company Law’s requirement that the proportion of independent directors must surpass one-third is essentially met by the sample listed companies. The minimum of BSIZE is 4, and the maximum is 17, which complies with the provisions of Chinese Company Law.

**TABLE 2:** Descriptive statistics.

Variables	N	Mean	S.D.	Min	P25	Median	P75	Max
FB	12,248	0.06	0.23	0.00	0.00	0.00	0.00	1.00
PAT	12,248	1.90	1.65	0.00	0.00	1.95	3.09	9.35
ROA	12,248	0.04	0.07	-0.34	0.02	0.04	0.07	0.21
LEV	12,248	0.39	0.19	0.06	0.24	0.38	0.53	0.86
SIZE	12,248	22.19	1.30	17.95	21.25	21.99	22.89	28.54
SOE	12,248	0.26	0.44	0.00	0.00	0.00	1.00	1.00
FAGE	12,248	2.95	0.30	1.39	2.77	3.00	3.18	4.14
INSHOLD	12,248	0.40	0.25	0.00	0.17	0.41	0.61	0.90
SALEGRO	12,248	0.17	0.34	-0.50	0.00	0.12	0.27	1.92
Tobin’s Q	12,248	1.89	1.11	0.84	1.23	1.55	2.10	7.56
IDRATIO	12,248	0.38	0.05	0.20	0.33	0.36	0.43	0.80
BSIZE	12,248	8.35	1.62	4.00	7.00	9.00	9.00	17.00

### 4.2. Pearson correlation analysis

Table 3 presents the correlation coefficient matrix among the variables. The variable correlation coefficient matrix shows that the correlation coefficient between FB and PAT is significant at the 1% level, revealing that CEO FB has a significant negative correlation with corporate innovation, supporting H1. Additionally, PAT has a significant positive correlation with ROA, SIZE, and SALEGRO. Further, PAT has a significant negative correlation, indicating that the selection of these variables into the empirical model is appropriate.

**TABLE 3:** Pearson correlation analysis.

	FB	PAT	ROA	LEV	SIZE	SOE	FAGE	INSHOLD	SALEGRO	Tobin’s Q	IDRATIO	BSIZE
FB	1											
PAT	−0.040***	1										
	(0.000)											
ROA	−0.035***	0.127***	1									
	(0.000)	(0.000)										
LEV	0.003	−0.004	−0.345***	1								
	(0.717)	(0.669)	(0.000)									
SIZE	0.007	0.039***	−0.013	0.529***	1							
	(0.459)	(0.000)	(0.162)	(0.000)								
SOE	−0.022**	−0.060***	−0.071***	0.272***	0.404***	1						
	(0.014)	(0.000)	(0.000)	(0.000)	(0.000)							
FAGE	0.026***	−0.092***	−0.078***	0.160***	0.196***	0.247***	1					
	(0.004)	(0.000)	(0.000)	(0.000)	(0.000)	(0.000)						
INSHOLD	−0.003	−0.033***	0.090***	0.175***	0.437***	0.413***	0.079***	1				
	(0.752)	(0.000)	(0.000)	(0.000)	(0.000)	(0.000)	(0.000)					
SALEGRO	−0.001	0.021**	0.280***	0.024***	0.033***	−0.056***	−0.092***	0.022**	1			
	(0.906)	(0.022)	(0.000)	(0.007)	(0.000)	(0.000)	(0.000)	(0.015)				
Tobin’s Q	0.024***	0.009	0.176***	−0.264***	−0.299***	−0.133***	−0.039***	−0.010	0.083***	1		
	(0.007)	(0.319)	(0.000)	(0.000)	(0.000)	(0.000)	(0.000)	(0.255)	(0.000)			
IDRATIO	0.015 (0.100)	0.003 (0.740)	−0.018* (0.052)	0.000 (0.984)	−0.022** (0.017)	−0.063*** (0.000)	−0.021** (0.021)	−0.077*** (0.000)	−0.011 (0.223)	0.043*** (0.000)	1	
BSIZE	−0.001	0.014	0.002	0.144***	0.299***	0.288***	0.118***	0.240***	−0.009	−0.102***	−0.548***	1
	(0.910)	(0.119)	(0.823)	(0.000)	(0.000)	(0.000)	(0.000)	(0.000)	(0.327)	(0.000)	(0.000)	

*p*-values in parentheses; *p < 0.10, **p < 0.05, and ***p < 0.01.

### 4.3. Main analysis

The results of the main regression analysis are reported in Table 4. The coefficient of FB in column (1) is −0.103, which is significantly negative at the 10% level (*t* = −1.72), indicating that CEO FB has a negative impact on firm innovation, and the results of the regression analysis support the H1. Column (2) reports the moderating effect of managerial ownership on the relationship between CEO FB and corporate innovation, and the coefficient of FB*MO is the primary concern. The coefficient of FB*MO in column (2) is 1.407 significant at the 1% level (*t* = 3.48), indicating that managerial ownership mitigates the negative effect of CEO FB on firm innovation, supporting H2.

**TABLE 4:** Regression results of the main analysis.

	(1)	(2)
	**PAT**	**PAT**
FB	−0.103*	−0.193***
	(−1.72)	(−2.87)
FB*MO		1.407***
		(3.48)
MO		0.527***
		(4.52)
ROA	2.919***	2.786***
	(14.37)	(13.65)
LEV	0.084	0.103
	(0.91)	(1.11)
SIZE	0.183***	0.186***
	(10.25)	(10.39)
SOE	−0.072**	−0.057
	(−1.90)	−1.49)
FAGE	−0.368***	−0.350***
	(−7.58)	(−7.17)
INSHOLD	−0.177***	−0.054
	(−2.82)	(−0.80)
SALEGRO	−0.147***	−0.153***
	(−3.56)	(−3.72)
Tobin’s Q	0.019	0.020
	(1.39)	(1.45)
IDRATIO	0.267	0.210
	(0.84)	(0.66)
BSIZE	0.053***	0.053***
	(4.48)	(4.52)
IND	yes	yes
YEAR	yes	yes
Constant	−3.766***	−3.940***
	(−8.56)	(−8.91)
*F*-value	79.63***	79.55***
Adj. R2	0.231	0.233
N	12,248	12,248

*t* statistics in parentheses; *p < 0.1, **p < 0.05, and ***p < 0.01; heteroskedasticity robust standard errors are used.

### 4.4. Robustness checks

#### 4.4.1. Robustness check using an alternative dependent measurement

We performed robustness tests using a modified corporate innovation measurement method. According to Faleye et al. (2014), we defined R&D intensity (RD) as a proxy variable for corporate innovation, which is the ratio of R&D expenditures to total assets; second, according to the patent law, all patents can be divided into three types: invention, utility model, and design patents. The invention patent refers to the proposal of a novel technical solution and might be the best indicator of a company’s capacity for innovation. Referring to Kong et al. (2021), we chose the number of invention patent applications for listed companies and then constructed another new dependent variable, PAT2, with the natural logarithm of the number of applications plus 1.

Table 5 shows the results of the robustness tests after changing the measure of corporate innovation. The coefficient of FB in column (1) is significantly negative at the 1% level (−0.003 with *t* = −5.13) and the coefficient of FB in column (3) is significantly negative at the 10% level (−0.087 with *t* = 1.74), revealing that CEO FB has a negative effect on corporate innovation after changing the dependent variable measurement. The coefficient of FB*MO in column (2) is positive and significant at the 5% level (0.011 with *t* = 2.18) and the coefficient of FB*MO in column (4) is positive and significant at the 1% level (1.101 with *t* = 3.04), indicating that managerial ownership mitigates the negative effect of CEO FB on firm innovation after replacing the dependent variable. The empirical results in Table 5 imply that H1 and H2 remained robust after changing the measurement method of corporate innovation.

**TABLE 5:** Results of robustness check using an alternative dependent measurement.

	(1)	(2)	(3)	(4)
	**RD**	**RD**	**PAT2**	**PAT2**
FB	−0.003***	−0.004***	−0.087*	−0.157***
	(−5.13)	(−5.83)	(−1.74)	(−2.84)
FB*MO		0.011**		1.101***
		(2.18)		(3.04)
MO		0.007***		0.481***
		(5.02)		(4.91)
ROA	0.018***	0.016***	1.996***	1.878***
	(5.29)	(4.79)	(11.84)	(11.09)
LEV	0.002	0.002*	0.039	0.056
	(1.49)	(1.68)	(0.49)	(0.70)
SIZE	−0.001***	−0.001***	0.218***	0.220***
	(−5.01)	(−4.84)	(13.61)	(13.74)
SOE	−0.002***	−0.002***	0.021	0.035
	(−3.93)	(−3.41)	(0.63)	(1.06)
FAGE	−0.004***	−0.003***	−0.297***	−0.280***
	(−6.24)	(−5.80)	(−7.17)	(−6.75)
INSHOLD	0.001	0.003***	−0.113**	−0.003
	(1.20)	(3.09)	(−2.11)	(−0.05)
SALEGRO	0.001*	0.001	−0.123***	−0.129***
	(1.70)	(1.56)	(−3.61)	(−3.78)
Tobin’s Q	0.003***	0.003***	0.044***	0.045***
	(13.25)	(13.29)	(3.64)	(3.72)
IDRATIO	−0.004	−0.005	0.032	−0.023
	(−1.32)	(−1.59)	(0.11)	(−0.08)
BSIZE	0.001	0.0002	0.034**	0.034**
	(1.54)	(1.56)	(3.25)	(3.28)
IND	yes	yes	yes	yes
YEAR	yes	yes	yes	yes
Constant	0.042***	0.040***	−4.438***	−4.595***
	(9.42)	(8.90)	(−11.19)	(−11.54)
*F*-value	203.68***	196.6***	66.46***	64.45***
Adj. *R*^2^	0.365	0.367	0.192	0.195
*N*	12,248	12,248	12,248	12,248

*t* statistics in parentheses; *p < 0.1, **p < 0.05, and ***p < 0.01; heteroskedasticity robust standard errors are used.

#### 4.4.2. Robustness check using additional control variables

We added CEO gender (GENDER), age (CEOAGE), and overseas background (OVERSEAS) to the control variables. Huang and Kisgen (2013) found that male executives exhibit more overconfidence in corporate decision-making, compared with female executives. GENDER is a dummy variable with a value of 1 if the CEO is male and 0 otherwise. CEOAGE indicates the age of the CEO in the current year; Musteen et al. (2006) argued that older CEOs tend to avoid risky innovations and resist reforms. OVERSEAS refers to CEOs with experience studying or working abroad. Yuan and Wen (2018) pointed out that both foreign study or work experience has a significant impact on corporate innovation.

Table 6 shows the results of the robustness tests after using additional control variables. The coefficient of FB in column (1) is negative and significant at the 10% level (−0.109 with *t* = −1.81), indicating that CEO FB still has a negative effect on corporate innovation after adding the control variables. The coefficient of FB*MO in column (2) is positive and significant at the 1% level (1.426 with *t* = 3.52), indicating that after increasing the control variables, managerial ownership mitigates CEO FB’s negative effect on firm innovation. The empirical results shown in Table 6 demonstrate that H1 and H2 remained robust after adding new control variables.

**TABLE 6:** Results of the robustness check using additional control variables.

	(1)	(2)
	**PAT**	**PAT**
FB	−0.109*	−0.202***
	(−1.81)	(−3.00)
FB*MO		1.426***
		(3.52)
MO		0.566***
		(4.78)
ROA	2.941***	2.807***
	(14.46)	(13.74)
LEV	0.092	0.111
	(0.99)	(1.19)
SIZE	0.182***	0.185***
	(10.21)	(10.38)
SOE	−0.066*	−0.049
	(−1.73)	(−1.28)
FAGE	−0.364***	−0.341***
	(−7.49)	(−6.99)
INSHOLD	−0.176***	−0.041
	(−2.80)	(−0.61)
SALEGRO	−0.150***	−0.156***
	(−3.62)	(−3.81)
Tobin’s Q	0.017	0.019
	(1.27)	(1.36)
IDRATIO	0.272	0.219
	(0.86)	(0.69)
BSIZE	0.054***	0.055***
	(4.53)	(4.61)
GENDER	−0.024	−0.024
	(−0.45)	(−0.46)
CEOAGE	−0.080	−0.188*
	(−0.86)	(−2.00)
OVERSEAS	0.089**	0.082*
	(2.09)	(1.94)
IND	yes	yes
YEAR	yes	yes
Constant	−3.438***	−3.235***
	(−6.14)	(−5.79)
*F*-value	76.03***	75.25***
Adj. *R*^2^	0.231	0.234
*N*	12,248	12,248

*t* statistics in parentheses; *p < 0.1, **p < 0.05, and ***p < 0.01; heteroskedasticity robust standard errors are used.

#### 4.4.3. Endogeneity test using propensity score matching

In the model, CEO FB is not affected by corporate innovation; accordingly, there was no endogeneity problem due to reverse causality in the model. The measurement of CEO FB in the model is accurate, accordingly, there was no endogeneity problem caused by variable measurement bias. Enterprises must be non-random when hiring CEOs, thus there may be endogeneity problems due to sample selection bias. Therefore, we used propensity score matching to alleviate any potential endogeneity problems due to sample selection bias, as follows. First, a sample of firms with CEOs who a background in finance was selected as the treatment group, whereas a sample of firms without such CEOs is selected as the control group. Second, CEO FB was selected as the independent variable, whereas LEV, SIZE, FAGE, SALEGRO, ROA, Tobin’s Q, SOE, INSHOLD, IDRATIO, and BSIZE were selected as covariates. After performing a logit regression to determine the propensity score, we utilized the one-to-four nearest neighbor matching approach to determine the final propensity score and one-to-four matching to minimize the mean square error (Abadie et al., 2004).

**FIGURE 1:**
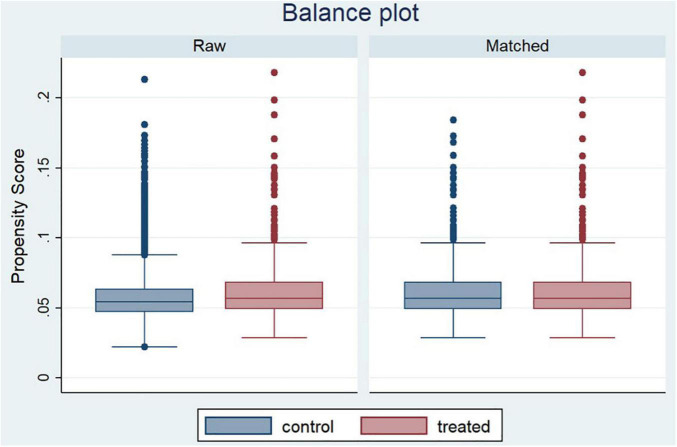
Box plot of covariates before and after matching.

Figure 1 shows a box plot of the covariates before and after matching to check for balance. The unmatched raw data is on the left. Between the treated and control groups, there are some obvious variations in the propensity scores. The outcome of the match is shown on the right. After matching, the covariates of the two groups are evenly distributed, indicating that the data are generally balanced.

The average effect on the treated (ATET) intervention group is reported in Table 7 after pairing with the control group using the propensity score. As presented in Table 7, the coefficient of FB is −3.37 significant at the 1% level, which validates that CEOs with FB have a negative effect on corporate innovation.

**TABLE 7:** Average treatment effect on the treated.

ATET	PAT	Coef.	AI robust std. err.	*z*	*P* > |z|	(95% conf. interval)	
	FB						
	(1vs0)	−0.2464	0.0731	−3.37***	0.001	−0.3897	−0.1031

***p < 0.01.

## 5. Conclusion and discussion

### 5.1. Conclusion

Sustainable development has become a key topic within the context of escalating global environmental issues. In this regard, studying how to foster corporate innovation is essential, from a practical standpoint, because it drives sustainable development. Based on imprinting theory, we analyzed data from Chinese A-share-listed companies for the period 2017–2021 to explore the relationship between CEO FB and corporate innovation. According to our research, CEOs with FB have a detrimental impact on the innovation of their enterprises. However, when CEOs have higher managerial ownership, this negative impact is mitigated. After a series of robustness tests, our findings remained valid. The mechanisms by which CEO background influences corporate micro behavior are analyzed in this study, thereby contributing to the literature on the variables influencing corporate innovation.

### 5.2. Theoretical contribution

First, while existing literature on the topic is relatively extensive, it is firmly rooted in upper-echelon theory and mainly attentive to the statistical relationship between CEO experience or background and corporate innovation. Notably, prior studies have overlooked this relationship’s intrinsic mechanisms. Based on imprinting theory, we provide an in-depth interpretation of the mechanisms by which CEO characteristics influence corporate innovation.

Second, prior research on CEO background has primarily explored their foreign (Yuan and Wen, 2018), military (Benmelech and Frydman, 2015), and academic experience (Shen et al., 2020), and paid little attention to their financial experience. This study incorporated CEO FB into the analytical framework of corporate innovation and added managerial ownership as a moderating variable to provide new empirical evidence for the study of factors influencing corporate innovation.

Third, several studies (Hirshleifer et al., 2012; Bromiley and Rau, 2016; Kashmiri et al., 2017) have examined this topic in the Western context; however, CEO cognition and behavior are deeply influenced by culture. Therefore, we explored the mechanisms from the perspective of Chinese culture, thus expanding the applicability of the relevant literature.

### 5.3. Practical implications

Based on the finding that CEO FB inhibits corporate innovation, enterprises should fully consider the compatibility between CEO background and the backgrounds of other executives, control the weight of executives with a background in financials to reduce the negative impact on corporate innovation strategy, and build innovation-oriented executive teams. Second, imprinting theory reveals that executive experience forms the imprint that affects their cognition and capabilities, which in turn impacts their decision-making; thus, enterprises should introduce an executive rotation system that encourages executives to abandon the rigid thinking answerable to their FBs, re-examine innovation strategies from new perspectives, and enhance their cognitive and decision-making abilities. Third, the findings suggest that managerial ownership inhibits the adverse effects of CEO FB on corporate innovation; in response, enterprises should establish and improve equity incentive plans to create incentive compatibility, thus allowing CEOs to exert their initiative and promote corporate value creation.

Regarding executives, first, they should be aware of how their background affects their behavior and decision-making, objectively perceive their own background imprinting and the limits of their reasoning, and fully take into account any background traits that could potentially influence their decision-making. Second, to establish the groundwork for scientific decision-making, executives should collaborate with team members from all backgrounds, gather information from various sources, and reduce their cognitive bias.

### 5.4. Limitations and future research directions

This study had a few limitations. First, due to limitations in the availability of research data, this study only included listed companies. Future research should also collect data from non-listed companies to test whether the FBs of CEOs in non-listed companies also impact corporate innovation.

Second, we mainly explored the impact of CEO FB on corporate innovation from the perspective of corporate technology innovation. However, in addition to technological innovation, corporate innovation also includes product innovation, process innovation, and other dimensions. Future studies should explore the relationship between CEO FB and product innovation or process innovation to specify the analytical framework of this topic.

Third, the national culture model developed by Hoftede et al. (2010) states that national cultural attributes vary. The national culture of a domestic firm influences its own internal culture and the consciousness of individuals in the firm, which forms idiosyncratic firm decisions and behavior. Therefore, we argue that investigating the connection between individual characteristics within firms and corporate behavior in various national cultural contexts is an important and valuable study path for future studies.

## Data availability statement

The original contributions presented in this study are included in the article/supplementary material, further inquiries can be directed to the corresponding author.

## Author contributions

YT has collected the data. YG and YT wrote the first draft. YG and JZ analyzed the data, contributed to the research problem formulation, theory, analysis, and conclusion. All authors listed have made a substantial, direct, and intellectual contribution to the work, and approved it for publication.
